# Supplementary figures and images for: Epithelial polarization in 3D matrix requires DDR1 signaling to regulate actomyosin contractility
Source: Life Sci Alliance. 2019 Feb 13;2(1):e201800276. doi: 10.26508/lsa.201800276 (PMC6374992; doi:10.26508/lsa.201800276)

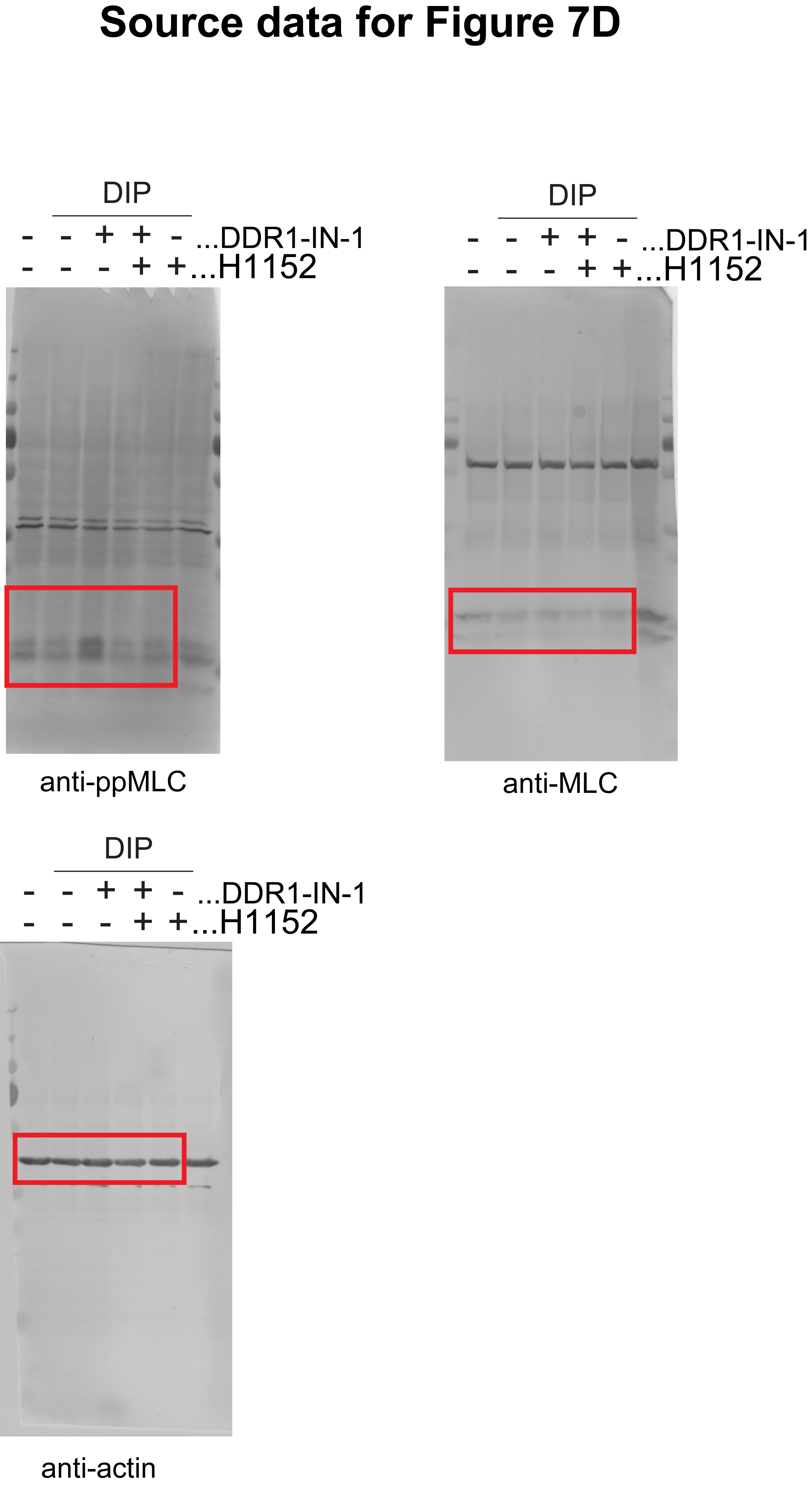

Supplement: Supplementary file 1 [file LSA-2018-00276_Sdata7.tif]
